# Socioeconomics inequities associated with different domains of physical activity: results of the National Health Survey 2019, Brazil

**DOI:** 10.1590/SS2237-9622202200015.especial

**Published:** 2022-08-01

**Authors:** Danielle Keylla Alencar Cruz, Kelly Samara da Silva, Marcus Vinicius Veber Lopes, Fernanda Ramos Parreira, Heitor Martins Pasquim

**Affiliations:** 1 Ministério da Saúde, Secretaria de Vigilância em Saúde, Brasília, DF, Brazil; 2 Universidade Federal de Santa Catarina, Programa de Pós-graduação em Educação Física, Florianópolis, SC, Brazil; 3 Universidade Federal de Goiás, Faculdade de Educação Física e Dança, Goiânia, GO, Brazil; 4 Universidade Federal de São Paulo, Departamento de Ciências do Movimento Humano, Santos, SP, Brazil

**Keywords:** Cross-Sectional Studies, Motor Activity, Public Health, Social Determinants of Health, Descriptive Epidemiology, Socioeconomic Factors

## Abstract

**Objective:**

To examine the socioeconomic indicators associated with engagement in physical activity (PA) in the leisure-time, transportation, domestic and occupational domains, in Brazilian adults.

**Methods:**

Cross-sectional study with secondary data from the National Health Survey (PNS), conducted in 2019. The factors associated with engagement in PA were analyzed using logistic regression.

**Results:**

The study involved 88,500 Brazilian adults with mean age of 45 ± 17.5 years old. Longer working hours [*odds ratio* (OR) = 0.74; 95%CI 0.66;0.82; > 40h *vs.* ≥ 20h] and female sex (OR = 0.67; 95%CI 0.63;0.71) were associated with lower chances of engaging in leisure-time PA. Higher income (OR = 3.20; 95%CI 2.79;3.67; > 5 *vs.* ≥ minimum wage) and education level (OR = 3.01; 95%CI 2.74;3.32 - complete higher education *vs.* incomplete elementary school) were positively associated with leisure-time PA.

**Conclusion:**

Socioeconomic correlates were strongly related to engagement in PA in Brazilian adults, suggesting a pattern of inequity marked by the need for survival, which is socially reproduced.

**Study contributions**

**Main results**

Longer working hours were associated with lower chances of engagement in physical activity (PA) in the domestic, transportation and leisure time domains, and with a higher probability of intense PA at work.

**Implications for services**

The findings of the study reveal the need to encourage engagement in PA during leisure time among the rural population, women and low-income individuals.

**Perspectives**

More studies on PA focused on health inequities should be encouraged to produce knowledge which is centered on health management decision-making, aiming at reducing inequities and guaranteeing the constitutional right to leisure.

## Introduction

Physical activity (PA) plays a fundamental role in daily life, being a source of social transformation and a potential strategy for health promotion.^1^ Thirteen of the 17 objectives proposed for sustainable development can be achieved by encouraging PA, which integrates the global health agenda with goals to be achieved by 2030.^2^ To this end, it is essential that teaching, research and politics adopt a holistic view of PA.^1^

With regard to inequity, a study analyzing data from 111 countries showed the existence of inequities in the distribution of PA within countries, and observed that a large part was due to the low level of PA among women. According to the authors, in cities where it was possible to walk, PA was higher throughout the day in all age, sex and body mass index groups.^3^ Another study,^4^ developed in the European context, pointed out that socioeconomic inequalities assert the situation of morbimortality resulting from non-communicable chronic diseases. In this case, leisure time PA was linked to income level and social class, that is, to the socioeconomic status, and was less prevalent than occupational PA.

In Brazil, social inequities are quite expressive and interfere with the health of the population.^5^ Data from the 2013 edition of the National Health Survey (PNS)^6^ already pointed to important social inequalities in Brazilian adults, such as greater physical inactivity during leisure time among the least educated, individuals of Non-White race/skin color and among those without a private health insurance. Therefore, the present study aimed to analyze the sociodemographic indicators associated with engagement in PA in leisure time, transportation, domestic and occupational domains, based on data from the 2019 PNS.

## Method

### Study design

This was a secondary analysis of data from the 2019 PNS, carried out by the Brazilian Institute of Geography and Statistics (IBGE) in partnership with the Ministry of Health. The microdata were accessed on December 1, 2020, based on their availability in the subdirectory of the IBGE webpage dedicated to the survey.^7^

### Population and sample

A three-stage cluster sampling design was used, as follows: (i) census tracts or set of sectors, as primary units; (ii) households, as secondary units; (iii) individuals aged ≥ 15 years, as tertiary units. Simple random sampling was adopted for all stages. Details regarding the sampling procedure are available in published documents.^8^ ^,^ ^9^ In this study, individuals age ≥ 18 years with complete data on all variables analyzed were considered eligible. Information was obtained through a household interview by means of mobile devices programmed with the survey questionnaire. The interviews were scheduled and conducted by previously trained interviewers on the dates which were most convenient for the respondents.

### Treatment of data

The dependent variables were: (i) domestic PA includes doing heavy cleaning, carrying loads or other heavy activities (does not consider paid domestic activity); (ii) PA during transport involves walking or cycling to work or other usual activities; (iii) PA during leisure time includes doing physical exercises or playing sports; (iv) PA at work covers walking, heavy cleaning, carrying heavy loads or other high intensity activities that require intense physical effort at work. The variables referring to each PA domain were dichotomized into “not engaged” when the participant reported not being involved in PA or being involved less frequently than once a week; and “engaged” when reporting engagement for at least once a week.

The type of PA most frequently reported by participants was engagement in leisure time PA. The analysis categories were: not engaged in leisure time PA; walking (outdoors/on a treadmill); running (outdoors/on a treadmill); cycling (bicycle/stationary bike); weight training/muscle strengthening (including localized gymnastics/pilates/stretching/yoga); gymnastics/aerobics (including spinning/step/jump/water aerobics/dancing); sports (swimming/martial arts and wrestling/soccer/basketball/volleyball/tennis). The questions related to PA in the PNS instrument were previously validated.^10^ ^,^ ^11^

The independent variables were: census status (urban; rural); *per capita* income (up to half the minimum wage - MW; more than half to one MW; more than one to two MWs; more than two to three MWs; more than three to five MWs; more than five MWs); education (incomplete elementary education, complete elementary education, complete high school, complete higher education); sex (male; female); age group (18 to 24; 25 to 39; 40 to 59; ≥ 60); race/skin color (White; Black; Brown); lives with a spouse (yes; no); work/employment status (out of the labor force; in the labor force and occupied; in the labor force and unoccupied) and working hours (up to 20 hours per week; from 21 to 30 hours per week; from 31 to 40 hours per week; more than 40 hours per week).

### Statistical analysis

The characteristics of the sample were described using relative frequencies. The factors associated with engagement in PA in the different domains were analyzed using binary logistic regression models (one model for each domain), considering participants not engaged in PA in the respective domain as a reference group. The analysis referring to the occupational domain included only the participants who were occupied. The association between sociodemographic indicators and the most frequent type of leisure time PA was analyzed using multinomial logistic regression, considering participants not engaged in leisure time PA as a reference group. In all models, the simultaneous entry of sociodemographic variables was adopted and the results were expressed as *odds ratios* (OR) and their respective 95% confidence intervals (95%CI).

Given that the independent variable referring to working hours is conditioned to the participants in the labor force, it was decided to combine the variables “work status/employment” and “working hours” in a single factor to estimate the indicators. The resulting variable was called “work status and working hours” and includes the following categories: working hours of up to 20 hours per week; 21 to 30 hours per week; 31 to 40 hours per week; more than 40 hours per week; out of the labor force; in the labor force and unoccupied. Collinearity was evaluated using Spearman's correlation matrix between sociodemographic variables and estimating the variance inflation factor for all models, on the basis of which no cases of multicollinearity were observed. The analyses were carried out using Stata software, version 16 (StataCorp, College Station, TX, USA), considering the complex cluster sampling design and incorporating the sample weights from the survey command.

### Ethical considerations

The PNS was approved by the National Committee for Ethics in Research, under the National Health Council, in August 2019, through opinion No. 3,529,376. Participation was voluntary, upon signature of the Free, Prior and Informed Consent Form.

## Results

Of the 94,114 households with residents aged ≥ 15 years, selected within the scope of the research, 90,846 were interviewed. Of those, 88,500 (53.2%; 95%CI 52.6;53.7 female individuals; mean age 45 ± 17.5 years) met the eligibility criteria and were analyzed. Sociodemographic information on the population can be seen in Table 1. It was found that 49.6% (95%CI 49.0;50.1) of the participants reported walking or cycling at least once a week. The prevalence of engagement in intense PA in the domestic context and at work was observed in 15.8% (95%CI 15.4;16.3) and 48.0% (95%CI 47.2;48.8) of the participants, respectively. In the context of leisure, 40.5% (95%CI 39.9;41.1) of the participants reported engaging in PA, highlighting walking 15.4% (95%CI 15.0;15.8), weight training 8.6% (95%CI 8.2;8.9) and sports 8.2% (95%CI 7.8;8.5) as the most frequent activities (Table 2).

**Table 1:** Characteristics of the participants age ≥ 18 years selected for the interview (n = 88,500), 2019 National Health Survey, Brazil

Variables	% (95%CI)^a^
**Census classification**	
Urban	86.2 (86.0;86.4)
Rural	13.8 (13.7;14.0)
***Per capita* income (minimum wages)**	
Up to 1/2	22.0 (21.6;22.5)
> 1/2 to 1	29.2 (28.7;29.7)
> 1 to 2	28.2 (27.6;28.7)
> 2 to 3	9.1 (8.7;9.4)
> 3 to 5	6.4 (6.1;6.6)
> 5	5.2 (4.9;5.4)
**Educaton**	
Incomplete elementary education	34.8 (34.2;35.3)
Complete elementary education	14.5 (14.1;14.9)
Complete high school	34.9 (34.4;35.5)
Complete higher education	15.8 (15.4;16.2)
Sex	
Male	46.9 (46.3;47.4)
Female	53.2 (52.6;53.7)
**Age groups (years)**	
18 to 24	13.9 (13.4;14.4)
25 to 39	29.2 (28.7;29.8)
40 to 59	35.3 (34.7;35.9)
≥ 60	21.6 (21.2;22.1)
**Race/skin color**	
White	43.3 (42.7;43.8)
Black	11.5 (11.1;11.8)
Brown	43.8 (43.2;44.4)
**Lives with a spouse**	
No	38.6 (38.0;39.2)
Yes	61.4 (60.8.0;62)
**Work/employment status**	
In the labor force and occupied	61.3 (60.7;61.8)
In the labor force and unoccupied	5.3 (5.0;5.6)
Out of the labor force	33.5 (32.9;34.0)
**Working hours** ^**b**^ **(hours per week)**	
Up to 20	12.9 (12.4;13.4)
21 to 30	10.5 (10.1;10.9)
31 to 40	33.1 (32.4;33.8)
> 40	43.5 (42.8;44.3)

a) 95%CI: 95% confidence interval; b) Data estimated in adults who were occupied in the reference week (n = 52,447).

**Table 2:** Engagement in PA prevalence equal to or greater than one day per week, in the domains of physical activity (PA) for Brazilian adults (n = 88,500), 2019 National Health Survey, Brazil

Variables	% (95%CI)^a^
**Domestic**	
No	84.2 (83.8;84.6)
Yes	15.8 (15.4;16.3)
**Occupational** ^**a**^	
No	52.0 (51.2;52.8)
Yes	48.0 (47.2;48.8)
**Transportation**	
No	50.4 (49.9;51.0)
Yes	49.6 (49.0;50.1)
**Leisure time**	
No	59.5 (58.9;60.1)
Yes	40.5 (39.9;41.1)
**Most frequent activity during leisure time**	
Does not engage in any	59.5 (58.9;60.1)
Walking/hiking	15.4 (15.0;15.8)
Running	2.2 (2.1;2.4)
Cycling	2.0 (1.8;2.2)
Weight training/muscle strengthening	8.6 (8.2;8.9)
Aerobics/academy gymnastics	3.2 (3.0;3.4)
Sports	8.2 (7.8;8.5)

a) 95%CI: 95% confidence interval; b) Data estimated in adults who were occupied in the reference week (n = 52,447).

Participants residing in rural areas were less likely to engage in PA in the transportation domain (OR = 0.67; 95%CI 0.63;0.71) and during leisure time (OR = 0.79; 95%CI 0.75; 0.85), and more likely to perform intense PA at work (OR = 1.61; 95%CI 1.49;1.74). Participants with higher income were more likely to engage in leisure time PA, and less likely to engage in PA in the transportation or occupational domains. Compared to males, females were more involved in domestic PA (OR = 2.85; 95%CI 2.63;3.08) and during transportation (OR = 1.21; 95%CI; 1.15;1.27), and less during leisure time (OR = 0.67; 95%CI 0.63;0.71) and at work (OR = 0.66; 95%CI 0.62;0.71). Participants of Black race/skin color were more likely to engage in domestic PA (OR = 1.15; 95%CI 1.02;1.28), during transportation (OR = 1.36; 95%CI 1.25;1.47) and at work (OR = 1.19; 95%CI 1.07;1.32) when compared to white individuals. It was found that longer working hours (> 40 hours per week) were associated with lower chances of engaging in domestic PA (OR = 0.71; 95%CI 0.62;0.80), during transportation (OR = 0.70; 95%CI 0.63;0.77) and during leisure time (OR = 0.74; 95%CI 0.66;0.82), and a greater chance of engaging in intense PA at work (OR = 1.71 ;95%CI 1.53;1.91) (Table 3).

**Table 3:** Associations between sociodemographic indicators and engagement in physical activity (PA) in the leisure time, transportation, domestic and occupational domains in Brazilian adults (n = 88,500), 2019 National Health Survey, Brazil

Variables	PA domains			
	Domestic	Transportation	Leisure time	Occupational^a^
	OR^b^ (95%CI)^c^	OR (95%CI)^c^	OR (95%CI)^c^	OR (95%CI)^c^
**Census classification**				
Urban	1.00	1.00	1.00	1.00
Rural	1.06 (0.97;1.14)	0.67 (0.63;0.71)	0.79 (0.75;0.85)	1.61 (1.49;1.74)
***Per capita* income (minimum wages)**				
Up to 1/2	1.00	1.00	1.00	1.00
> 1/2 to 1	0.95 (0.86;1.04)	0.77 (0.72;0.83)	1.24 (1.15;1.34)	0.85 (0.78;0.94)
> 1 to 2	1.03 (0.92;1.14)	0.61 (0.57;0.66)	1.58 (1.45;1.71)	0.79 (0.72;0.88)
> 2 to 3	0.99 (0.85;1.15)	0.54 (0.49;0.60)	2.09 (1.87;2.33)	0.71 (0.62;0.81)
> 3 to 5	0.94 (0.79;1.13)	0.54 (0.48;0.61)	2.39 (2.11;2.70)	0.61 (0.52;0.71)
> 5	0.67 (0.55;0.81)	0.51 (0.45;0.58)	3.20 (2.79;3.67)	0.54 (0.45;0.63)
**Education**				
Incomplete elementary education	1.00	1.00	1.00	1.00
Complete elementary education	1.31 (1.18;1.46)	0.99 (0.92;1.07)	1.40 (1.28;1.52)	0.86 (0.77;0.95)
Complete high school	1.32 (1.21;1.45)	1.01 (0.94;1.07)	1.95 (1.82;2.09)	0.62 (0.56;0.67)
Complete higher education	1.21 (1.07;1.38)	0.84 (0.76;0.92)	3.01 (2.74;3.32)	0.38 (0.33;0.42)
Sex				
Male	1.00	1.00	1.00	1.00
Female	2.85 (2.63;3.08)	1.21 (1.15;1.27)	0.67 (0.63;0.71)	0.66 (0.62;0.71)
**Age group (years)**				
18 to 24	1.00	1.00	1.00	1.00
25 to 39	1.51 (1.32;1.71)	0.88 (0.80;0.96)	0.73 (0.67;0.80)	1.09 (0.97;1.24)
40 to 59	1.57 (1.38;1.78)	0.96 (0.87;1.05)	0.61 (0.56;0.67)	1.10 (0.97;1.24)
≥ 60	0.88 (0.76;1.02)	0.90 (0.82;1.00)	0.43 (0.39;0.48)	0.89 (0.76;1.04)
**Race/skin color**				
White	1.00	1.00	1.00	1.00
Black	1.15 (1.02;1.28)	1.36 (1.25;1.47)	0.99 (0.91;1.08)	1.19 (1.07;1.32)
Brown	0.99 (0.92;1.07)	1.11 (1.05;1.17)	1.01 (0.96;1.07)	1.04 (0.96;1.12)
**Lives with a spouse**				
No	1.00	1.00	1.00	1.00
Yes	1.34 (1.25;1.44)	0.79 (0.75;0.83)	0.91 (0.86;0.96)	1.01 (0.95;1.09)
**Work status and working hours (hours per week)**				
Up to 20	1.00	1.00	1.00	1.00
21 to 30	0.82 (0.70;0.96)	0.90 (0.80;1.02)	0.95 (0.84;1.08)	1.34 (1.17;1.52)
31 to 40	0.72 (0.63;0.82)	0.72 (0.65;0.80)	0.82 (0.74;0.91)	1.41 (1.26;1.58)
> 40	0.71 (0.62;0.80)	0.70 (0.63;0.77)	0.74 (0.66;0.82)	1.71 (1.53;1.91)
Out of the labor force	0.73 (0.65;0.82)	0.44 (0.40;0.49)	0.87 (0.79;0.97)	-
In the labor force and unoccupied	1.26 (1.06;1.49)	0.70 (0.60;0.80)	0.97 (0.83;1.12)	-

a) Analysis restricted to occupied adults in the reference week (n = 52,477); b) OR: **Odds ratio** obtained from the binary logistic regression adjusted simultaneously for all the variables presented in the table, with of not engaged in physical activity (PA) in the domain in question as reference; c) 95%CI: 95% confidence interval.

The associations between sociodemographic indicators and the most frequent types of leisure time PA are shown in Table 4. Participants who lived in rural areas were more likely to practice sports (OR = 1.24; 95%CI 1.10;1.38) and less likely to choose other activities. Higher income levels were associated with greater engagement in all types of PA, most notably weight training (OR = 8.65; 95%CI 6.93;10.79), gymnastics (OR = 4.54; 95%CI 3.21;6.42), and running (OR = 5.22; 95%CI 3.59;7.58). Females chose walking (OR = 1.12; 95%CI 1.04;1.21) and aerobic gymnastics (OR = 3.90; 95%CI 3.08;4.95) more often, and were less inclined to engage in running (OR = 0.29; 95%CI 0.24;0.35), cycling (OR = 0.30; 95%CI 0.24;0.37), and sports (OR = 0.08; 95%CI 0.06;0.09).

**Table 4:** Associations between sociodemographic indicators and most frequent types of leisure time physical activity (PA) among Brazilian adults (n = 88,500), 2019 National Health Survey, Brazil

Variables	Walking	Running	Cycling	Weight/Muscle training	Aerobics	Sports
	OR^a^ (95%CI)^b^	OR (95%CI)^b^	OR (95%CI)^b^	OR (95%CI)^b^	OR (95%CI)^b^	OR (95%CI)^b^
**Census classification**						
Urban	1.00	1.00	1.00	1.00	1.00	1.00
Rural	0.82 (0.76;0.90)	0.41 (0.30;0.55)	0.49 (0.36;0.68)	0.46 (0.39;0.55)	0.37 (0.29;0.47)	1.24 (1.10;1.38)
***Per capita* income (minimum wage)**						
Up to 1/2	1.00	1.00	1.00	1.00	1.00	1.00
> 1/2 to 1	1.29 (1.17;1.42)	1.20 (0.90;1.60)	1.31 (0.82;2.08)	1.66 (1.40;1.97)	1.33 (1.05;1.68)	0.88 (0.77;1.01)
> 1 to 2	1.54 (1.39;1.72)	1.81 (1.37;2.38)	1.42 (0.88;2.29)	2.67 (2.25;3.17)	2.02 (1.59;2.56)	0.94 (0.80;1.10)
> 2 to 3	1.85 (1.60;2.13)	2.46 (1.78;3.40)	1.52 (0.88;2.63)	4.26 (3.48;5.21)	3.43 (2.38;4.93)	1.03 (0.83;1.29)
> 3 to 5	2.05 (1.74;2.41)	3.12 (2.14;4.55)	1.91 (1.03;3.54)	5.07 (4.10;6.27)	3.55 (2.54;4.96)	1.16 (0.88;1.53)
> 5	2.11 (1.74;2.56)	5.22 (3.59;7.58)	2.39 (1.30;4.39)	8.65 (6.93;10.79)	4.54 (3.21;6.42)	1.54 (1.16;2.04)
**Education**						
Incomplete elementary education	1.00	1.00	1.00	1.00	1.00	1.00
Complete elementary education	1.20 (1.07;1.34)	2.09 (1.45;3.01)	1.48 (1.13;1.94)	1.73 (1.41;2.11)	1.20 (0.91;1.60)	1.81 (1.55;2.11)
Complete high school	1.71 (1.56;1.87)	4.53 (3.39;6.07)	1.81 (1.36;2.41)	3.32 (2.84;3.87)	1.83 (1.43;2.34)	2.13 (1.85;2.45)
Complete higher education	2.24 (1.98;2.55)	8.75 (6.35;12.05)	2.29 (1.64;3.19)	6.04 (5.07;7.20)	2.73 (2.06;3.62)	2.93 (2.40;3.59)
**Sex**						
Male	1.00	1.00	1.00	1.00	1.00	1.00
Female	1.12 (1.04;1.21)	0.29 (0.24;0.35)	0.30 (0.24;0.37)	1.01 (0.91;1.12)	3.90 (3.08;4.95)	0.08 (0.06;0.09)
**Age group (years)**						
18 to 24	1.00	1.00	1.00	1.00	1.00	1.00
25 to 39	1.19 (1.00;1.42)	0.72 (0.56;0.94)	0.83 (0.58;1.20)	0.75 (0.64;0.87)	0.81 (0.64;1.04)	0.44 (0.38;0.51)
40 to 59	1.86 (1.57;2.21)	0.45 (0.34;0.58)	0.70 (0.51;0.97)	0.41 (0.35;0.48)	0.81 (0.63;1.04)	0.17 (0.14;0.20)
≥ 60	1.44 (1.20;1.73)	0.14 (0.09;0.22)	0.65 (0.43;0.97)	0.25 (0.21;0.30)	0.68 (0.50;0.92)	0.04 (0.03;0.05)
**Race/skin color**						
White	1.00	1.00	1.00	1.00	1.00	1.00
Black	0.94 (0.83;1.05)	1.00 (0.77;1.29)	0.67 (0.48;0.93)	0.96 (0.81;1.12)	1.04 (0.81;1.33)	1.33 (1.14;1.55)
Brown	1.04 (0.97;1.13)	1.06 (0.89;1.27)	0.66 (0.51;0.84)	0.95 (0.86;1.06)	1.11 (0.94;1.31)	1.14 (1.02;1.29)
**Lives with a spouse**						
No	1.00	1.00	1.00	1.00	1.00	1.00
Yes	1.06 (0.99;1.14)	0.98 (0.82;1.18)	1.23 (1.00;1.51)	0.70 (0.64;0.77)	1.02 (0.89;1.17)	0.94 (0.84;1.05)
**Work status and working hours (hours per week)**						
Up to 20	1.00	1.00	1.00	1.00	1.00	1.00
21 to 30	0.90 (0.76;1.06)	1.15 (0.78;1.69)	0.74 (0.49;1.13)	1.06 (0.85;1.32)	1.05 (0.74;1.50)	0.83 (0.62;1.09)
31 to 40	0.67 (0.59;0.77)	1.17 (0.85;1.61)	0.83 (0.57;1.21)	0.94 (0.78;1.13)	0.89 (0.67;1.18)	0.81 (0.64;1.02)
> 40	0.65 (0.57;0.75)	0.98 (0.72;1.33)	0.66 (0.48;0.92)	0.85 (0.71;1.02)	0.76 (0.56;1.04)	0.70 (0.55;0.88)
Out of the labor force	0.95 (0.84;1.07)	0.94 (0.67;1.31)	0.60 (0.40;0.90)	0.87 (0.72;1.04)	1.03 (0.78;1.37)	0.62 (0.48;0.80)
In the labor force and unoccupied	1.12 (0.92;1.35)	1.25 (0.82;1.90)	0.43 (0.27;0.68)	1.09 (0.84;1.41)	1.03 (0.65;1.63)	0.74 (0.55;0.99)

a) OR: *Odds ratio* of the multinomial logistic regression adjusted simultaneously for all the variables presented in the table, the reference being not engaged in physical activity (PA) during leisure time; b) 95%CI: 95% confidence interval.

## Discussion

It is possible to identify multiple sociodemographic correlates for engagement in PA and PA typology in the investigated subgroups, when considering the domains of practice. Longer working hours, Black race/skin color, female sex, lower *per capita* income and level of education were associated with lower chances of engaging in leisure time PA. However, in this article, it is relevant to discuss the critical processes that are expressed in the association between sociodemographic indicators and the profile of PA engagement in the Brazilian population.

Given what has been pointed out, it can be observed that engagement in PA in Brazil is marked by socioeconomic inequities, which are the causes that account for the lower involvement in PA during leisure time by some groups and their higher level of engagement in activities in the occupational, transportation, and/or domestic domains, thus attesting a pattern of inequity that is socially reproduced, and which goes against what the Global Action Plan on PA advocates.^2^

It is important to point out that some ways of working and living are more harmful than others^12^ and that health determinants can be external to the health care and treatment system. To put it another way, social inequities generate health inequities.^13^ This understanding is necessary for a critical epidemiology with the potential to be an important tool for monitoring, developing public health awareness and planning public actions aimed at protecting the health of the population. To do so, it is essential to deconstruct infertile obstacles between different currents of epidemiological thought, especially those erected between the traditions of classical epidemiology and social epidemiology.^14^

Structural determinants linked to the conflict between capital and work reverberate in the singular dimension of engagement in PA. In this sense, the discussion on the promotion of PA must take into account the tendency for time expropriation and recognize that, in a capitalist society, human activity will always face a fierce and unequal dispute over the subjects' available time.^15^ Therefore, this reflection cannot be separated from the right to leisure, health, non-degrading work and the objective living conditions that enable choices that are favorable to health.

The study identified a revealing association of this labor-capital conflict. Occupied individuals who are subjected to longer working hours are less involved in leisure time PA. This is due to the fact that part of the worker's time and energy is consumed by the labor activity.^16^ The findings also show an association between a low prevalence of leisure time PA and a high prevalence in the occupational domain in rural residents. These results are not random and can be attributed to the long working hours, the lack of incentives (support from the family, neighborhood) and the lack of appropriate space for the practice of PA.^17^ ^,^ ^18^

An interesting issue unveiled by the study, with the potential to guide future research, is the significant volume of intense PA in the occupational domain in the Brazilian adult population. The rural population is more likely to have this concentrated volume during work, which would characterize an imbalance with demonstrably negative impacts on health.^19^ Researchers have observed that while engaging in leisure time PA is a choice made by the individual and which involves short durations of dynamic activities, with adequate resting time and consolidated health benefits, the opposite is observed for occupational PA.^20^

This context requires measures that seek collective health awareness of the impact of PA in the different domains on rural health and, at the same time, that equip policymakers so that they can promote engagement in leisure time PA. To this end, it is necessary to strengthen discussions and justified claims, such as a reduction in working years for retirement, a reduction in working hours without cutting down wages, and an increase in resting time, along with ergonomic adjustments in the production environment and processes, in addition to programs that promote the dissemination and access to spaces for PA in rural areas.

Health inequities are not restricted to the health-disease process, but integrate other inequities in terms of access to and use of health services. A cross-sectional study with data from the 2013 PNS^21^ corroborates this understanding by evidencing that knowledge of public PA programs increased according to income, but those who participated in public PA programs more often were individuals with lower income. 

However, some current forms of PA promotion reproduce inequalities by favoring socially and economically privileged groups.^22^ In recent years, the field of public health has shown greater interest in reflections and studies on social differentiation and inequity. This interest is expressed in public health policies, as is the case of the National Health Promotion Policy (PNPS).^23^ Thus, in order to corroborate the understanding of the health-disease process in social groups which were invisible until then, themes such as class, sex, sexuality and ethnicity are given prominence, in an intersectional manner.

Women’s greater engagement in physical activities in the domestic context is representative of the social role shaped by the sexual division of labor, anchored in patriarchal capitalism.^24^ Furthermore, the construction of femininity permeates the social hierarchy through biological aspects and the reproductive function, which generate, by means of work relationships, processes of domination and exploitation.^25^

This differentiation process was highlighted by the type of leisure time PA chosen. Women tend to go walking and do aerobic gymnastics, while men engage in activities such as weight training and sports. These findings corroborate the analysis carried out in the 2013 PNS,^26^ which showed that the practice of sports was less frequent among females. Another point identified in the 2013 PNS, and reinforced in the findings of the present study, was the continued engagement of females (18.4% in 2013 and 21.8% in 2019) compared to males (5.4% in 2013 and 9.1% in 2019) in PA in the domestic domain.^27^

The ways of acting and behaving permeate relations of power and domination. Therefore, the construction of bodies must be considered as a political construction^28^ and it is reflected in the way masculinity and femininity are understood and reproduced. Playing a sport is developed by reproducing behaviors which are considered acceptable and appropriate for either men or women, such as aggressiveness, passivity, domination, submission, virility and fragility.^29^

Despite the unquestionable advances in the organization of a network of PA programs, the linear and pragmatic approach of the field still preponderantly points to a physical-sanitary, regulatory, healthcare and medicalizing rationale. PA is presumed to be the necessary remedy for the prevention of diseases and suffering that are socially reproduced, and it brings with it an understanding that reinforces the individualizing and blaming process, which sometimes generates isolated, episodic actions and strategies, which are and disconnected from the reality of the territories and their populations.

The notion of care is imbricated in the power relations between subjects, which standardizes and regulates the use of bodies.^30^ Therefore, the maintenance of a hierarchical and vertical rationale, from planning and managing to executing programs, projects and actions focusing on PA, corroborates a discriminatory and excluding social dynamics. Therefore, there is an urgent need for a broader view of the concept of the benefits of PA that goes beyond the dominant justifications restricted to disease management and traditional physical health goals.

As for the limitations of the study, we emphasize that households located in special census sectors or with scarcely populated, such as indigenous and quilombola clusters, prisons and waterborne vessels, were excluded from the research sample. Consequently, some subgroups which are relevant to understand health inequities were not represented. Although this paper congregates researchers from different theoretical backgrounds, the approach undertaken does not enable a dialectical analysis that can explain the origin of each inequity revealed. Even so, it was possible to identify, quantify and discuss a series of processes of inequity in health that deserve specific studies.

The present research analyzed the sociodemographic indicators associated with engagement in PA in the different domains, revealing socioeconomic inequities for the domains of PA, due to which some groups are less likely to engage in PA during leisure time and more likely to do so in the occupational, transportation and/or domestic domains. This confirms a pattern of inequity that is socially reproduced, as inequalities arising from unfair social relations, which are avoidable, are evidenced, resulting in damage to health and life.

This pattern of inequity in the PA engagement profile was demonstrated in the subgroups with lower *per capita* income, people with longer working hours, those residing in rural areas, as well as women and individuals of Black race/skin color.

In this sense, the construction of public policies, programs and intersectoral and inter-institutional actions, especially those aimed at promoting PA, supported by the principles and guidelines of the Brazilian National Health (SUS), in a universal, equitable, participatory way and with a focus on social justice, need to consider aspects of social and sex differentiation, understanding that spaces aimed at promoting PA are sexed and socially hierarchical places.
